# Remarks on a Review of an Essay on Trembles, Milk-sickness, &c.

**Published:** 1842-01

**Authors:** John S. Seaton


### Art. VII.—*Remarks on a Review of an Essay on Trembles, Milk-sickness, &c.* By John S. Seaton, M. D.

It will be recollected by the readers of this Journal, that the November No. contains a review of a small pamphlet published by me during the past summer, on the cause of "Milk-sickness, Trembles, &c." If Dr. Drake, the author of that critique, had done justice to my views therein expressed, or if all who read the Journal had an opportunity of reading my pamphlet likewise, I would feel willing to let the whole pass without publicly noticing the criticism. But neither being the case, I feel myself called on to reply to Dr. Drake, and defend my expressed proposition.

I have said that the critic failed to mete out justice to the views contained in the pamphlet on the cause of the above named diseases, and I may add, as at least strong negative proof in support of this general charge, that the verdict of every man, professional and non-professional, with whom I have conversed, and who had read both the pamphlet and the review, is in full and entire coincidence with this declaration.

But a general charge cannot be sustained without specific items, and for this reason I will particularize a few out of many points in the criticism that are at variance with justice, both to myself and to the subject, in order to establish what I have said.

And 1. Dr. Drake did not disclose the reasons that I gave, for not having subjected the water of the different springs, etc., that I visited, to chemical analysis.

2. Dr. D. conveys the idea, that I assume the ground that *arsenical iron pyrites* is the universal cause of the disease, an opinion not only not advanced by me, but expressly denied on page 41 of the pamphlet. The reason why I spoke so freely and fully of the Iron, and especially of the sulphuret or pyrites, was because I found it in such great abundance in those sections, and also because of the ready and rapid spontaneous decomposition of the pyrites containing the arsenic.

3. The Professor at least intimated very plainly, that I had declared the "Milk-sickness" to be common to *all* sand or free-stone localities, which is not the fact, as he himself will see, if he will but trouble himself enough to read my pamphlet attentively, and give to my expressions their legitimate import or meaning. On page 10, I argued and averred, that the "*pure* and *thorough* limestone regions were exempt," while the regions containing the *impure* orders of lime-stone, mixed with sand-stone, or the almost entire sand-stone formation, were the ones in which the diseases abound to the greatest extent. This, however, neither affirms nor implies, that the diseases in question are present in *all* such localities in the South and West, and to read it in such sense, does violence both to my language and my meaning. But in the regions where those diseases prevail, I again assert, (Dr. D.'s assertions to the contrary, nevertheless,) that such is their geological constitution; while in the exclusive or entirely *pure* limestone lands, not containing beds of diluvial deposition, or any of the enumerated materials characteristic of the "coal measure," the diseases in question never have occurred, and never will occur, either *endemically*, or *sporadically*, unless they be transported. In such, for instance, as that portion of Jefferson County above Louisville, known as the "Bear-Grass settlement,' a goodly portion of Shelby, Fayette, and Bourbon Counties in Kentucky, and in the *pure blue* limestone around Cincinnati, Ohio, with innumerable other localities which might be mentioned, those diseases never did, and never will, appear. We might as reasonably, and with about as fair a chance of success, expect to find a gold mine in the public streets of Louisville or Cincinnati, or mountains of snow, and icebergs, in the centre of the Torrid Zone, as to expect to find "Milk-sickness" in such situations.

But 4. The most astonishing feature in the "Criticism" is contained in the paragraph in which Dr. Drake denies the above premise in the arsenical hypothesis, and endeavors to annihilate by the fiat of his own word, the geological distinctions laid down in the pamphlet.

With due and ample respect and regard for Dr. Drake's erudite attainments, and a prudent reverence for his respectable (even venerable) age, candor, truth, justice, and the subject in hand, compel me to say, that by this open and unqualified denial, and by referring to the different rivers in Kentucky, Ohio, and Indiana, which he has done, he has proved himself to be, either utterly ignorant of the subject on which he wrote, or totally regardless of, and recreant to the truth. I am prepared to say, and to establish, (if indeed many of as good men as live in Kentucky dare be believed,) that *sandstone* exists in abundance on the Kentucky river, at many points, but especially a short distance below Boonsborough, and at or near the mouth of Eagle creek, below Frankfort, and with it also is found the spurious or impure limestone. The same thing is true of many places on the Licking river, while abundance of good pure limestone exists in many portions on each river.

In relation to "all the Western tributaries of the Scioto river, in Ohio," I ask no better proof against the Professor's allegation in his review, than is to be found in the "Memoir on Milksickness," from his own pen in March last. Why did Dr. Drake in so short a time contradict what he said under the "Caption" on page 6th of the "Memoir"? Which statement is true, himself being umpire? In many places on the Miamis, the sandstone is abundant, being imbedded in a sandy soil, which is underlaid by a deep bed of diluvium, consisting of pebbles, sand, gravel, and clay, etc. This I state upon the authority of three citizens of this county, of unimpeachable veracity, and whose names I am at liberty to give, if necessary. But we will examine the stony formation in the bottoms of those rivers, and see how far it comports with the premise in the "Arsenical Hypothesis." Beginning at Columbus on the Scioto, in Franklin County, we find the river flowing over a stratum of "Cliff limestone" which is very impure, because of the magnesia, alumina, sand, and the abundance of sulphur and iron in the form of *pyrites* contained in it. At Chillicothe, in Ross County, it traverses the *black shale*, which overlays the "cliff limestone," and which also contains the pyrites in abundance. At Waverly, in Pike County, it runs on the fine grained sandstone, (which is remarkable because of the quantity of sulphur and iron it contains), and at its mouth is found on the coarse sandstone, which constitutes the base of the coal formation.

The Miami rivers, above Clifton on the one side and Troy on the other, are flowing over the "cliff limestone," and in that region the "milk-sickness" abounds; but below those towns the rivers flow over the *pure blue limestone*, and there the disease is unknown. At Brookville, on White-water, in Indiana, the blue limestone occurs; but it is there covered to the depth of many feet by a thick bed of diluvial or transported materials, consisting of clay, sand, gravel, and an immensity of the fragments of "cliff limestone."

In Preble county, situated on the Western border of Ohio, and through which several of the Western tributaries of the greater Miami pass, the "milk-sickness" prevails where the "cliff limestone" is covered to the depth of 40 feet, by the same kind of diluvium, &c. For the accuracy of the above statements, see "Geological report on the State of Ohio."

Such stony formation under-laying the streams which bound and pass through the "Virginia military district," such soil and timber growth, as is described by Dr. Drake, at the top, and growing on the surface of the plateaus or table lands, at once assure us, in the absence of all other testimony, of the presence of *inferior* limestone, sandstone, etc., etc., with more or less mineral substances.

If the latter be not true, then it is not a fact that geology and botany have a reciprocal relation. But we have other and ample testimony, and that, too, of the most "indubitable" character, equally as "*indubitable*" as the testimony upon which Dr. D. relies so confidently, in support of the *fact*, relative to the *fate* of the herd of "cattle," a part of which were pastured on *meadowed* land, while the others were kept upon an adjoining "wood land," &c.

But the professor assures us that the clearing and culture of the land has proved to be an *infallible preventive* in the State of *Ohio*, and adds: "We know of no fact going to prove that the same process would not be effectual every where." It may be true that Dr. D. *knows* of no fact going to prove the contrary, for I apprehend he has not tested the matter by observing for himself, and as nothing can properly be denominated *knowledge*, that has not been conveyed to the mind by one or more of the five senses. But I think a sufficiency of *facts* of at least a *veritable* aspect have been presented, contradictory of the Doctor's assumption, to induce *faith* in him of an opposite kind, if he were not *indeed* a thorough sceptic on the subject. If he will only turn to page 90 in the first number of the ninth volume of "Transylvania Journal of Medicine" he will see that his assertion relative to the *entire* efficiency of clearing and tilling the land in Ohio, as a means of prevention, was contradicted by Dr. McAnelly, of Ky., five years before its promulgation; and on page 87 of the same number he will find it again contradicted by Dr. Shelton, of Alabama, relative to *cultivated* land in Tennessee and Indiana. And if the settlers in the places to be named, can be accredited, it has not been sufficient on Beaver creek and Palloca, Indiana, in a small section below Hardinsburg, in Breckinridge, and on Panther creek, in Davis county, Ky., &c.

It may be true that too much attention has been bestowed on the cause of the *diseases*, but it is yet as interesting and of as much importance to the profession, as well as the people, as it was in *March last*, and surely it merits a much graver consideration than does another disease common to cattle, and which finds place occasionally in our Western periodicals, the *Murrain*.

In conclusion, I will add that since the publication of my pamphlet, I have possessed myself of many valuable facts by experimenting and otherwise, and while the matter is in hand, and the investigation pending, I am willing to meet any man in the west, in any periodical, and defend the "*Arsenical* Hypothesis," against the whole vegetable kingdom. I believe the arsenic is contained in spring water, in some instances, in *licks* in others, and is often exhaled from the earth in dry weather, and settles on the herbage; perhaps it rises in the form of *Arsenuretted Hydrogen*, is decomposed by the air, and the arsenic thrown down, &c. But the facts are what we want; and will any opponent of the *Arsenical Hypothesis* join issue on equal terms and oppose it before the reading public?

Dec. 30, 1841.

